# Evaluation of honey authenticity in Lebanon by analysis of carbon stable isotope ratio using elemental analyzer and liquid chromatography coupled to isotope ratio mass spectrometry

**DOI:** 10.1002/jms.4730

**Published:** 2021-05-17

**Authors:** Khaled El Hawari, Mohamad Al Iskandarani, Farouk Jaber, Raed Ezzeddine, Luca Ziller, Matteo Perini, Luana Bontempo, Maura Pellegrini, Federica Camin

**Affiliations:** ^1^ Laboratory for Analysis of Organic Compounds (LAOC) CNRSL, Lebanese Atomic Energy Commission (LAEC) Beirut Lebanon; ^2^ Faculty of Public Health I Lebanese University Beirut Lebanon; ^3^ Analysis of Organic Compounds Laboratory (LACO), Faculty of Sciences I Lebanese University Beirut Lebanon; ^4^ Department of Food Quality and Nutrition, Research and Innovation Centre Fondazione Edmund Mach (FEM) San Michele all'Adige Italy; ^5^ Isotope Mass Spectrometry and High Resolution Elemental Analysis Thermo Fisher Scientific S.P.A. Rodano Italy; ^6^ Center Agriculture Food Environment (C3A) University of Trento San Michele all'Adige Italy

**Keywords:** authenticity, carbon isotope ratio (^13^C/^12^C), EA‐IRMS, honey, LC‐IRMS

## Abstract

Honey is one of the most valuable sweeteners consumed by humans all over the world. Consequently, it is often a target for adulteration through the addition of different sugar syrups during or after honey production, resulting in a reduction in its nutritive value. For the first time, this study analyzes honey samples of various botanical species collected from different Lebanese regions using element analyzer (EA) and liquid chromatography (LC) coupled with isotope ratio mass spectrometry (IRMS). The δ^13^C of bulk honey, its protein fraction, and the main individual sugars (glucose, fructose, disaccharides, and trisaccharide) were determined, in order to characterize and evaluate the authenticity of honey consumed in Lebanon. The results showed that the δ^13^C values for bulk honey and its protein range from −26.5‰ to −24.5‰ and from −26.4‰ to −24.7‰, respectively, for authentic samples. δ^13^C values for samples adulterated with sugar syrups range from −11.2‰ to −25.1‰ for bulk honey and from −26.6‰ to −23.7‰ for its proteins, with a difference between bulk and protein values between −1 and −8.7‰. Using LC‐C‐IRMS techniques, the δ^13^C of individual sugars provides additional information on the presence of undeclared sugars. We found that all authentic samples had Δδ^13^C_f‐g_ and Δδ^13^C max values within the naturally occurring range of ±1‰ and ±2.1‰, respectively, while the adulterated samples fall outside the Δδ^13^C ranges. The oligosaccharide peak was detected in most adulterated samples.

## 1 INTRODUCTION

Honey is a natural complex product produced by *Apis mellifera* bees from nectar of plants, as well as from honey dew.^1^, ^2^ It is mainly composed of high amount of sugars (glucose, fructose, and sucrose) and small amounts of various constituents such as mineral salts, proteins, organic acids, enzymes, vitamins, pigments, phenolics, flavonoids, and volatile compounds.^3^ The composition of honey depends on several factors, such as the type of the nectar, bee species, geographical origin, seasons, mode of storage, and harvesting technology,^4^, ^5^ which can affect its quality value. Due to worldwide demand and high price of honey, beekeepers are triggered to adulterate their honey with cheaper sweeteners and additives.

The European Union Council Directive 2001/110/EC^2^ and FAO/WHO Codex Alimentarius^6^ prohibit the addition of any food ingredient or additive, as well as removal of any other substance except where this is unavoidable in the removal of foreign inorganic or organic matter. Despite that, there are several methods of adulteration of honey that could be practiced during its production; the most popular method involves the addition of sugar syrups (starch syrup and inverted syrup) directly into pure honey (direct adulteration) or by means of feeding bee colonies with different sugar syrups during honey production in winter when nectar is scarce (indirect adulteration).^7^ Mixing high with low quality honey is another adulteration practice used by beekeepers for economic reasons to increase the volume of honey.^8^

In Lebanon, honey is considered an important breakfast ingredient. The presence of different altitudes and the Mediterranean weather ensure a flowering season almost all over the year. According to Lebanon's Beekeeper's Association, Lebanon produces different types of honey between 18,000 and 20,000 t from 274,390 hives; most of them are intended for Lebanese consumption and partly exported from Lebanon to Arab countries and to Japan.^9^ Addam et al.^9^ reported that 58% of honey consumers in Lebanon check if their honey is adulterated or pure; 75% do not trust honey labels claiming that it is 100% natural; and 40% prefer to purchase honey directly from beekeepers. To the best of our knowledge, there is no previous published work regarding honey authenticity consumed in Lebanon. This problem can present a major obstacle to assess properly its quality in the markets.

Many analytical procedures are designed to assess the quality of honey samples. They include the analysis of sugar content through high‐performance liquid chromatography (HPLC),^10^ gas chromatography (GC),^11^ Fourier transform infrared spectroscopy (FTIS),^12^ and high‐performance anion exchange chromatography with pulsed amperometric (HPAED‐PAD).^13^ However, these techniques are not capable of identifying low levels of added sugars unambiguously, nor are they adequate to detect a sophisticated adulteration.^14^ On the other hand, the use of stable carbon isotope ratio analysis (SCIRA) using elemental analyzer‐isotope ratio mass spectrometry (EA‐IRMS) has opened new perspectives for food authenticity identification. This method can differentiate carbon isotope ratios ^13^C/^12^C that are produced by different photosynthesis cycles (C4 and C3 plants). In general, plants with the Calvin Benson photosynthetic cycle (C3 plants), which are resources for honey, have δ^13^C values ranging from −21‰ to −32‰. On the other hand, plants with the Hatch‐Slack photosynthetic cycle (C4 plants) like corn and cane syrup have δ^13^C values ranging from −9‰ to −18‰. This distinction makes it possible to detect honey adulteration with sugar syrups that originate from C4 plants. Natural honey is expected to have δ^13^C values ranging from −23.5‰ and −27.5‰ with average −25.4‰.^15^ When honey samples have δ^13^C values more positive than −23.5‰, this indicates a possible addition of exogenous sugars from cane or corn. However, Anklam^16^ reported that δ^13^C values alone are not suitable to prove adulteration of sugar syrups (C4 plants) in bulk honey samples; therefore, a SCIRA method was improved by measuring δ^13^C of bulk honey and its protein fraction, which serves as an internal standard. The δ^13^C of protein and the sugars of honey should be the same if they come from the same source, but when exogenous sugars were supplied into pure honey, the δ^13^C values of honey will change while δ^13^C values of protein fraction will not be affected; thus, the difference in δ^13^C values between these two fractions will increase. There is a general agreement that the minimum difference in δ^13^C between honey sample and its associated protein extract should not be more than 1‰ for pure honey.^15^ A difference higher than 1‰ indicates that the honey is adulterated. This approach was adopted worldwide and it is considered as a reference method to detect minimum 7% of C4 sugar.^17^, ^18^

Unfortunately, AOAC 998.12 method is not suitable to determine the authenticity of honey containing low amount of protein (like acacia or lavender), or high amount of yeast,^19^ or when raw honey is treated by ultrafiltration membranes to eliminate microorganisms and proteins,^20^ or in the case of authentic citrus honey where the difference between the δ^13^C value of honey and protein (Δδ^13^C_p‐h_) always exceed 1‰, indicating its adulteration with high fructose corn syrup (C4 sugar %) more than 7% according to the AOAC method.^21^ Moreover, adulteration of honey with other types of sugar syrups that originate from C3 plants (like beet and rice sugar) is not detectable by using AOAC 998.12 method.^22^ Elflein and Raezke^19^ reported that these drawbacks could be solved by using liquid chromatography coupled to isotope ratio mass spectrometry (LC‐IRMS). This technique can separate individual sugar constituents of honey (e.g., glucose, fructose, and sucrose) and determine their δ^13^C values simultaneously. Various studies of honey authenticity have previously been conducted using LC‐IRMS.^14^, ^19^, ^22^, ^23^, ^24^, ^25^, ^26^, ^27^, ^28^, ^29^ They demonstrate that the measured δ^13^C values of sugars as well as the natural occurring differences between the δ^13^C values of these sugars (Δδ^13^C) yield to define authenticity criteria that detect adulteration of honey with both C4 and C3 sugars with a sensitivity of 1% to 10%.^19^, ^22^

The present work aims to evaluate the authenticity of honey from various botanical species, collected directly from beekeepers from different Lebanese area and from local market using EA‐IRMS and LC‐IRMS. The δ^13^C of bulk honey and its protein fraction and δ^13^C of the main individual sugars (glucose, fructose, sucrose, trisaccharide, and oligosaccharide) were determined and assessed using authenticity criteria described in AOAC 998.12 ^17^ method and by Elflein and Raezke.^19^ The results obtained in this work could be used as input data for fraud detection in Lebanese honey in order to minimize a potential food risk for the consumer.

## 2 MATERIALS AND METHODS

### 2.1 Reagents and standards

Sugars standards (D‐(+)‐glucose, D‐(−)‐fructose, sucrose) at purity higher than 99% were purchased from Sigma Aldrich (Milano, Italy). Crystalline phosphoric acid (>99%) and sodium peroxodisulfate (>99%) were purchased from Honeywell (Charlotte, North Carolina, USA). Sodium tungstate dihydrate analytical grade was purchased from Fluka (Honeywell) and sulfuric acid analytical grade was purchased from Honeywell. Deionized water with resistivity 18.2 Ωm was prepared with arium® comfort Water purification systems from Sartorius (Sartorius AG, Goettingen, Germany).

Sucrose IAEA‐CH6 (δ^13^C value: −10.449‰) and fuel oil NBS‐22 (δ^13^C value: −30.031‰) were purchased from IAEA (International Atomic Energy Agency, Vienna, Austria) and used as reference standards for carbon stable isotope measurement on EA‐IRMS and LC‐IRMS.

### 2.2 Honey samples

Thirty‐three honey samples were collected directly from local beekeepers from different regions (22 samples) and from local Lebanese markets (11 samples) between September 2019 and January 2020. The botanical origins of the samples were different and shown in Table 1. The samples were received at the laboratory in glass container and kept in the dark at room temperature until the time of their analysis.

**TABLE 1:** δ^13^C values (‰) of honeys and their protein fractions in honey samples measured by EA‐IRMS

Sample no.	Floral	Origin	δ^13^C_h_	δ^13^C_p_	Δδ^13^C_p‐h_	C4 sugar (%)	Honey quality
S1	Oak and thyme	Market	−19.1 ± 0.06	−24.7 ± 0.03	−5.6	37.4	Adulterated
S2	Wild flowers	Market	−23.4 ± 0.07	−25.1 ± 0.07	−1.7	11.2	Adulterated
S3	Thyme and Thistles	Market	−16.0 ± 0.03	−23.7 ± 0.06	−7.7	54.8	Adulterated
S4	Orange Blossom	Market	−13.5 ± 0.05	‐	‐	‐	Adulterated
S5	Oak and thyme	Market	−18.6 ± 0.04	−25.6 ± 0.06	−7.0	44.0	Adulterated
S6	Thyme and Thistles	Market	−19.2 ± 0.04	−26.0 ± 0.03	−6.8	41.5	Adulterated
S7	Aspen trees	Beekeepers	−25.0 ± 0.05	−25.0 ± 0.01	0.0	−0.1	Pure
S8	Oak	Beekeepers	−26.4 ± 0.02	−26.3 ± 0.03	0.1	−0.9	Pure
S9	Thyme	Beekeepers	−24.7 ± 0.02	−25.1 ± 0.04	−0.4	2.9	Pure
S10	Apiaceae	Beekeepers	−25.3 ± 0.01	−26.0 ± 0.02	−0.7	4.5	Pure
S11	Oak	Beekeepers	−25.8 ± 0.01	−25.6 ± 0.03	0.2	−1.1	Pure
S12	Summer	Beekeepers	−24.5 ± 0.02	−24.9 ± 0.03	−0.3	2.2	Pure
S13	Citrus	Beekeepers	−25.2 ± 0.03	−26.2 ± 0.03	−1.0	7.0	Pure
S14	Oak	Beekeepers	−26.5 ± 0.05	−26.4 ± 0.04	0.1	−0.6	Pure
S15	Oak	Beekeepers	−25.1 ± 0.03	−26.6 ± 0.02	−1.5	8.6	Pure
S16	Summer	Beekeepers	−25.4 ± 0.12	−25.1 ± 0.01	0.3	−1.6	Pure
S17	Pine	Beekeepers	−24.7 ± 0.04	−25.5 ± 0.02	−0.8	5.1	Pure
S18	Oak	Beekeepers	−24.9 ± 0.01	−25.6 ± 0.05	−0.7	4.5	Pure
S19	Oak	Beekeepers	−24.1 ± 0.05	−25.9 ± 0.04	−1.8	11.1	Adulterated
S20	Oak	Beekeepers	−23.7 ± 0.01	−25.8 ± 0.03	−2.1	13.2	Adulterated
S21	Summer	Beekeepers	−24.5 ± 0.07	−25.3 ± 0.02	−0.8	4.9	Pure
S22	Summer	Beekeepers	−25.4 ± 0.05	−25.7 ± 0.02	−0.3	1.7	Pure
S23	Oak	Beekeepers	−25.2 ± 0.02	−25.8 ± 0.03	−0.6	3.7	Pure
S24	Citrus	Beekeepers	−25.0 ± 0.01	−25.8 ± 0.06	−0.7	4.7	Pure
S25	Summer	Beekeepers	−25.2 ± 0.01	−25.7 ± 0.03	−0.4	2.6	Pure
S26	Citrus	Beekeepers	−25.0 ± 0.01	−25.5 ± 0.02	−0.5	3.0	Pure
S27	Chandab and loquat	Beekeepers	−24.8 ± 0.02	−25.1 ± 0.02	0.3	−1.6	Pure
S28	Summer	Beekeepers	−22.4 ± 0.05	−24.4 ± 0.06	−2.0	13.4	Adulterated
S29	Wild flowers	Market	−24.9 ± 0.06	−24.7 ± 0.02	0.2	−1.3	Pure
S30	Orange Blossom	Market	−22.7 ± 0.05	−25.0 ± 0.02	−2.3	14.9	Adulterated
S31	Multifloral	Market	−11.2 ± 0.07	‐	‐	‐	Adulterated
S32	Wild flowers	Market	−23.6 ± 0.03	−24.7 ± 0.04	−1.0	7.0	Pure
S33	Black forest	Market	−23.8 ± 0.06	−25.5 ± 0.06	−1.7	11.0	Adulterated

Abbreviations: δ^13^C_h_, δ^13^C of bulk honey; δ^13^C_p_, δ^13^C of protein; Δδ^13^C_h‐p_, difference between δ^13^C of bulk honey and apparent C4 sugar content calculated according to AOAC method 998.12.

### 2.3 Sample preparation

For EA‐IRMS analysis, AOAC official method AOAC 998.12 was adopted to determine δ^13^C in bulk honey and its proteins. In brief, 1 mg of honey was weighed in a small tin capsule using a microbalance and placed into the autosampler of the elemental analyzer. As for protein analysis, 10 g of honey were weighed into a 50 ml disposable centrifuge tube. Then 4 ml of distilled water were added to dissolve the honey. After shaking, 2 ml of sodium tungstate (10% in aqueous solution) and sulfuric acid (0.335 M) was added into the centrifuge tube. The sample was shaken and then incubated in a water bath at 80°C until a visible floc forms with a clear supernatant. Following that, the tubes were filled with distilled water and centrifuged for 5 min at 4000 rpm. The supernatant was removed and the precipitated protein was washed with distilled water and centrifuged. The wash is repeated four times until the supernatant was clear. The precipitated protein was placed into a freeze dryer for 1 day. Then 0.5 mg approximately of the protein was placed into tin capsules.

For LC‐IRMS analysis, 150 mg of honey was diluted by adding 100 ml of ultrapure water and filtered through a 0.2 μm PVDF membrane filter (Millipore, France). Finally, the filtered sample was transferred into LC vials for analysis.

### 2.4 Instrumentation and measurement

#### 2.4.1 EA‐IRMS analysis

The analysis of δ^13^C of bulk honey and its protein fraction were performed in triplicates using an IRMS (visION, Isoprime Ltd, UK) coupled with an elemental analyzer (Vario Isotope Cube, Elementar Analysensysteme GmbH, Germany). The sample in tin capsule was combusted at 1175°C in a reactor packed with tungsten (VI) oxide. The oxides byproducts were removed in a reduction reactor containing reduced copper at 630°C. The carrier gas (He) flow was 125 ml/min. The δ^13^C values bulk honey and its protein fraction were calculated against in‐house protein working standards which were calibrated against international reference standards IAEA‐CH6 and NBS‐22. The results of δ^13^C were accepted if the standard deviation of the three measurements was less than 0.1‰. The isotope ratios were expressed in δ‰ against Vienna‐Pee Dee Belemnite according to the following equation:

$$\mathrm{\delta iE}=\frac{{\mathrm{iR}}_{\mathrm{SA}}-{\mathrm{iR}}_{\mathrm{REF}}}{{\mathrm{iR}}_{\mathrm{REF}}}$$

where R_SA_ is the isotope ratio measured for the sample and R_REF_ is the international standard isotope ratio.

#### 2.4.2 LC‐IRMS analysis

The chromatographic system consisting of an HPLC system (UltiMate 3000, Dionex, Thermo Scientific, Germany) was coupled to a Delta V plus isotope ratio mass spectrometer (Thermo Scientific, Germany) via LC isolink interface. The analysis was conducted on an RCM‐monosaccharide Ca^2+^ column (300 × 7.8 mm; 8 μm) obtained from Phenomenex and heated at 55°C. The eluent 100% ultrapure water was used at a flow rate of 0.3 ml/min to separate individual sugars in honey. The total running time was 45 min and the injection volume was 10 μl. Once the analytes elute from the column, they pass through the LC isolink interface where the oxidation reagents, 0.5 M sodium peroxodisulfate and 1.7 M phosphoric acid, were mixed to the mobile LC phase at a flow rate of 20 μl/min to produce CO_2_ inside the oxidation reactor at 99.8°C.

**FIGURE 1:**
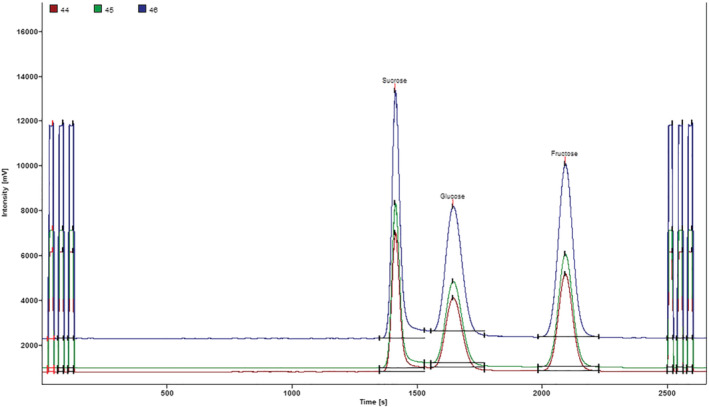
Typical chromatograms for sugars standard mixture by LC‐IRMS

All samples were analyzed at least three times and the mean value for δ^13^C was adopted. A mixture of sugars standard solution containing fructose, glucose and sucrose prepared in water at 0.8 g/L was used as working in‐house standard to determine δ^13^C values of individual sugars in honey (Figure 1). The δ^13^C values of pure single sugar for this mixture were determined previously with EA‐IRMS against international reference standards IAEA‐CH6 and NBS‐22. The values of δ^13^C of sucrose, glucose and fructose obtained from EA‐IRMS are −12.3 ± 0.02‰, −20.2 ± 0.02‰, and −26.5 ± 0.05‰, respectively. Those sugars were then used as working standards for LC‐IRMS measurements.

## 3 RESULTS AND DISCUSSION

### 3.1 Uncertainty of δ^13^C EA‐IRMS and LC‐IRMS measurements

To assess the method performance for the determination of the carbon stable isotope ratio in honey and its protein fraction by EA‐IRMS as well as its individual sugars by LC‐IRMS, a quality control pure honey sample was analyzed on different days within 4 months. The standard deviation of intra‐laboratory reproducibility (SD_R_) was assessed and the results are shown in Table 2. We can observe that SD_R_ values for δ^13^C of honey and protein were 0.1‰ and 0.2‰, respectively, by using EA‐IRMS. These values are lower to those indicated in AOAC official method 998.12.^17^ The SD_R_ obtained for glucose, fructose and disaccharide were within the same range as described by Elflein and Raezke^19^ and Cabañero et al.^22^ and by the results of an interlaboratory comparison recently published by the Joint research council (European commission)^30^ (SD_R_ < 0.3‰). For trisaccharides, the SD_R_ obtained by LC‐IRMS was higher (SD_R_ = 0.6‰) due to their low amounts present in honey and to the limitation of the HPLC column in separating individual trisaccharides, resulting in broad and overlapped peak shapes. In terms of uncertainty (U = 2xSD_R_), we can observe that uncertainty of δ^13^C values using EA‐IRMS were 0.2‰ for bulk honey, 0.4‰ for its proteins and 1.8% for the apparent C4 sugar. Regarding the LC‐IRMS analysis, the uncertainty of δ^13^C values for δ^13^C_glucose,_ δ^13^C_fructose_, Δδ^13^C_f‐g_ (differences between δ^13^C fructose and δ^13^C glucose) and Δδ^13^C max (the maximum difference between all possible measured δ^13^C values) was 0.4‰, for δ^13^C_disaccharides_ was 0.6‰ and for δ^13^C_trisaccharide_ was 1.2‰.

**TABLE 2:** δ^13^C precision results of EA‐IRMS and LC‐IRMS for quality control honey sample (n = 6)

	Mean	SD_R_	U = 2 SD_R_
δ^13^C_honey (h)_ (‰)	−26.4	0.1	0.2
δ^13^C_protein (p)_ (‰)	−26.5	0.2	0.4
δ^13^C_fructose (f)_ (‰)	−26.1	0.2	0.4
δ^13^C_glucose (g)_ (‰)	−26.2	0.2	0.4
δ^13^C_disaccharide (ds)_ (‰)	−27.4	0.3	0.6
δ^13^C_trisaccharides (ts)_ (‰)	−25.5	0.6	1.2
Δδ^13^C_p‐h_ (‰)	0.003	0.1	0.2
C4 Sugar (%)	−0.02	0.9	1.8
Δδ^13^C_f‐g_ (‰)	0.1	0.2	0.4
Δδ^13^C_f‐ds (_‰)	1.3	0.3	0.6
Δδ^13^C_f‐ts_ (‰)	−0.5	0.4	0.8
Δδ^13^C_f‐p_ (‰)	0.1	0.3	0.6
Δδ^13^C_g‐ds_ (‰)	1.2	0.2	0.4
Δδ^13^C_g‐ts_ (‰)	−0.6	0.3	0.6
Δδ^13^C_g‐p_ (‰)	0.03	0.3	0.6
Δδ^13^C_ds‐ts_ (‰)	−1.8	0.3	0.6
Δδ^13^C_ds‐p_ (‰)	−1.2	0.3	0.6
Δδ^13^C_ts‐p_ (‰)	0.7	0.5	1.0
Δδ^13^C max (‰)	1.9	0.2	0.4

Abbreviations: n, number of measurements on different days; SD_R_, standard deviation of intra‐laboratory reproducibility; U, uncertainty (2× SD_R_).

### 3.2 δ^13^C analysis of honey and its protein by EA‐IRMS

Honey samples and their protein fraction were analyzed by EA‐IRMS to determine carbon stable isotopic composition. As shown in Table 1, δ^13^C values were ranged from −26.5‰ to −11.2‰ for bulk honey (δ^13^C_h_) and from −26.6‰ to −23.7‰ for the extracted protein (δ^13^C_p_). The standard deviation for δ^13^C_h_ and δ^13^C_p_ values from three triplicates was less than 0.1‰. In our study, we used two authenticity criteria to distinguish between authentic and adulterated honey samples. The first one is described by White and Winters based on δ^13^C_h_, which is more positive than −23.5‰ for adulterated honey^15^ and the second one is through calculating the difference (Δδ^13^C_p‐h_) between the δ^13^C_p_ and δ^13^C_h_. Honey is considered to be adulterated if the difference Δδ^13^C_p‐h_ exceed −1‰ which correspond to 7% adulteration with C4 sugar.^17^ The apparent C4 sugar content was calculated according to equation: C4 sugar (%) = [(δ^13^C_p_ − δ^13^C_h_) × 100] ÷ [δ^13^C_p_ − (−9.7)].^17^ The measurement uncertainty will be taken into account when stating compliance with the authenticity criteria.

The results in Table 1 show that 21 out of 33 honey samples were classified as authentic, because δ^13^C_h_ was more negative than −23.5‰^15^ and the Δδ^13^C_p‐h_ was below −1‰.^17^ Four of the adulterated samples (S2, S19, S20, and S33) resulted authentic according to White and Winters^15^ but exceeded the Δδ^13^C_p‐h_ acceptable limit of −1‰ with apparent C4 sugar ranging from 11% and 13.2%. Regarding samples S4 and S31, which are honeys with non‐extractable proteins, only δ^13^C for bulk honey could be measured at −13.5‰ and −11.2‰, respectively. Therefore, they were classified as adulterated.

### 3.3 δ^13^C analysis of honey by LC‐IRMS

The LC‐IRMS method was used to determine carbon stable isotope ratio of individual sugars (δ^13^C glucose (δ^13^C_g_), δ^13^C fructose (δ^13^C_f_), δ^13^C disaccharide (δ^13^C_ds_), and δ^13^C trisaccharide (δ^13^C_ts_) present in 33 honey samples. In our study, we adopted authenticity criteria reported by Elflein and Raezke^19^ to detect honey adulteration with exogenous sugars, taking into consideration the associated uncertainties. This approach was based on measuring δ^13^C for individual sugars in honey and then calculating the differences between these values (Δδ^13^C) and between δ^13^C values of the isolated protein (δ^13^C_p_). The limits for Δδ^13^C values of authentic honey were the following: Δδ^13^C max and Δδ^13^C_f‐g_ should be within ±2.1‰ and ±1‰, respectively. Furthermore, Elflein and Raezke reported that the oligosaccharide peak should not be detected at high concentration (above 0.7 area%) in authentic honeys where it indicates the presence of exogenous sugar.

**FIGURE 2:**
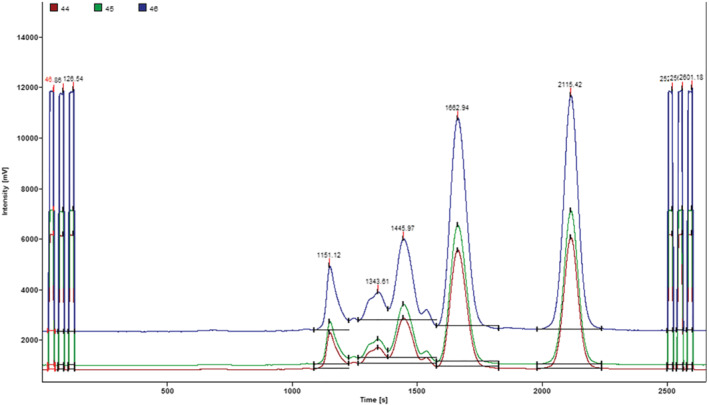
Chromatogram for adulterated honey (sample S3 in Table 1), δ^13^C oligosaccharides (RT: 1151s): −12.6‰, δ^13^C trisaccharides (RT: 1343s): −11.9‰, δ^13^C disaccharides (RT: 1445s): −13.2‰, δ^13^C glucose (RT: 1662s): −15.7‰, and δ^13^C fructose (RT: 2115 s): −17.3‰

The δ^13^C values for fructose, glucose, disaccharides, and trisaccharides (shown in Table 3) ranged from −27.2‰ to −13.9‰, from −27.2‰ to −12.3‰, from −28.0‰ to −12.5‰, from −28.3‰ to −11.3‰, respectively. The standard deviations (SDs) for individual sugars (n = 3 per each) were as follows: 0.01–0.44‰ for δ^13^C_f_, 0.02–0.67‰ for δ^13^C_g,_ 0.03–0.49‰ for δ^13^C_ds_, and 0.01–1‰ for δ^13^C_ts_. These SDs values were within the standard deviation ranges reported by Elflein and Raezke. The obtained δ^13^C values show clearly that some honey samples were adulterated based on both inappropriate chromatoghraphic profile of sugars (Figure 2) and on stable isotope ratios.

**TABLE 3:** δ^13^C values (‰) of the main sugars and their differences Δ δ^13^C in honey samples measured by LC‐IRMS

Sample no.	δ^13^C_f_	δ^13^C_g_	δ^13^C_ds_	δ^13^C_ts_	Δδ^13^C_f‐g_	Δδ^13^C max	Os (area%)	Honey quality
S1	−22.2 ± 0.3	−19.3 ± 0.3	−17.7 ± 0.2	−13.1 ± 0.2	−2.9	11.6	2.1	Adulterated
S2	−24.5 ± 0.4	−23.2 ± 0.4	−22.3 ± 0.4	−16.6 ± 0.1	−1.3	8.5	2.1	Adulterated
S3	−17.3 ± 0.3	−15.7 ± 0.1	−13.2 ± 0.2	−11.9 ± 0.1	−1.7	11.8	6.3	Adulterated
S4	−16.3 ± 0.1	−13.8 ± 0.2	−12.5 ± 0.2	−11.9 ± 0.3	−2.6	4.4	10.8	Adulterated
S5	−21 ± 0.3	−18.1 ± 0.1	−15.4 ± 0.1	−12.4 ± 0.2	−2.9	13.2	7.5	Adulterated
S6	−22.1 ± 0.1	−18.8 ± 0	−16.5 ± 0.3	−13.7 ± 0	−3.3	12.2	6.1	Adulterated
S7	−25.2 ± 0.3	−24.7 ± 0.2	−26 ± 0.3	ND	−0.4	1.2	<0.7	Pure
S8	−27.2 ± 0.3	−27.2 ± 0	−28 ± 0.3	−27.3 ± 0.3	−0.1	2.1	ND	Pure
S9	−26.4 ± 0.2	−26 ± 0.3	−26.6 ± 0.1	−26.3 ± 0.8	−0.4	1.5	<0.7	Pure
S10	−25.5 ± 0.2	−25.4 ± 0	−26.1 ± 0	−26.8 ± 0.4	−0.1	1.4	<0.7	Pure
S11	−24.6 ± 0.1	−24.1 ± 0.2	−25.2 ± 0.3	−24.3 ± 0.3	−0.5	1.5	<0.7	Pure
S12	−25.2 ± 0.4	−25.3 ± 0.4	−25.5 ± 0.4	−24.7 ± 0.2	0.1	0.8	<0.7	Pure
S13	−24.8 ± 0.2	−25.3 ± 0.2	−25.8 ± 0.2	−24.1 ± 0.1	0.5	2.1	<0.7	Pure
S14	−24.5 ± 0.1	−24.5 ± 0.2	−26.4 ± 0.2	−26.4 ± 0.1	0.0	1.9	ND	Pure
S15	−24.7 ± 0.4	−23.8 ± 0.7	−25 ± 0.4	−23.7 ± 0.2	−1.0	2.8	<0.7	Adulterated
S16	−25.3 ± 0	−25.6 ± 0	−25.4 ± 0	−24.2 ± 0.2	0.3	1.4	<0.7	Pure
S17	−24.9 ± 0.1	−24.4 ± 0.2	−26.1 ± 0.4	−25.1 ± 1	−0.4	1.6	ND	Pure
S18	−24.9 ± 0	−24.7 ± 0.1	−25.1 ± 0.5	−26.1 ± 0.3	−0.2	1.4	ND	Pure
S19	−23.3 ± 0.2	−22.9 ± 0.1	−24.8 ± 0.3	−26.6 ± 0.1	−0.4	3.6	5.3	Adulterated
S20	−23 ± 0.2	−22.6 ± 0.3	−24 ± 0.2	−25.9 ± 0	−0.4	3.3	7.5	Adulterated
S21	−24.6 ± 0.1	−24.5 ± 0.2	−26.1 ± 0.3	−25.1 ± 0	−0.1	1.7	ND	Pure
S22	−25.7 ± 0	−26 ± 0.2	−25.4 ± 0.3	−24.7 ± 0.3	0.3	1.3	ND	Pure
S23	−25.2 ± 0.1	−25.2 ± 0.1	−26.7 ± 0.3	−26.9 ± 0.3	−0.1	1.8	ND	Pure
S24	−25.2 ± 0.2	−25.6 ± 0	−25.8 ± 0.1	−24.7 ± 0	0.4	1.1	ND	Pure
S25	−25.6 ± 0.2	−25.6 ± 0.1	−25.6 ± 0.1	−24 ± 0.2	0.0	1.7	ND	Pure
S26	−25.5 ± 0.2	−25.5 ± 0.3	−26 ± 0.4	−25.6 ± 0.1	0.0	0.5	<0.7	Pure
S27	−24 ± 0.1	−24 ± 0.2	−24.6 ± 0.2	−25.9 ± 0.1	0.0	2.0	<0.7	Pure
S28	−22 ± 0.2	−22.1 ± 0.1	−21.6 ± 0.1	−17 ± 0.4	0.1	7.4	<0.7	Adulterated
S29	−25.7 ± 0.1	−25.6 ± 0.1	−26.6 ± 0.5	ND	−0.1	1.9	ND	Pure
S30	−22.1 ± 0.1	−22.9 ± 0.3	−22.2 ± 0.2	−22 ± 0.1	0.8	2.9	ND	Adulterated
S31	−13.9 ± 0.1	−12.3 ± 0.2	−13 ± 0.2	−11.3 ± 0.1	−1.6	2.6	42.0	Adulterated
S32	−23.9 ± 0.1	−25.3 ± 0.1	−25.7 ± 0.3	−23.5 ± 0	1.3	2.3	ND	Pure
S33	−22.6 ± 0.2	−23.1 ± 0.1	−24.2 ± 0.3	−23.1 ± 0.1	0.6	2.9	ND	Adulterated

Abbreviations: δ^13^C_f_, δ^13^C of fructose; δ^13^C_g_, δ^13^C of glucose; δ^13^C_d_, δ^13^C of disaccharides; δ^13^C_ts_, δ^13^C of trisaccharides; Δδ^13^C_f‐g_, differences between δ^13^C of fructose and δ^13^ of glucose; Δδ^13^C max, maximum absolute differences between all δ^13^C values including δ^13^C of protein; Os (Area%), area of oligosaccharide chromatographic peak in %.

**FIGURE 3:**
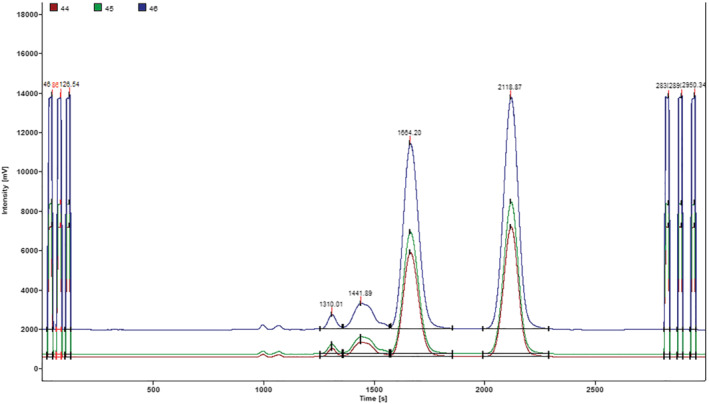
Chromatogram for authentic honey (sample S22 in Table 1), δ^13^C trisaccharides (RT: 1310s): −24.67‰, δ^13^C disaccharides (RT: 1441s): −25.42‰, δ^13^C glucose (RT: 1664s): −25.98‰, and δ^13^C fructose (RT: 2118 s): −25.72‰

Twenty honey samples with δ^13^C values for fructose, glucose, disaccharides, and trisaccharides within the expected ranges reported by and Raezke,^19^ Dong et al.,^24^ and Kawashima et al.^26^ were considered authentic. As reported in Table 3, the δ^13^C values of these authentic honeys ranged from −27.23‰ to −23.94‰ for δ^13^C_f_, from −27.16‰ to −23.96‰ for δ^13^C_g,_ from −28.03‰ to −24.55‰ for δ^13^C_ds,_ from −27.32‰ to −23.47‰ for δ^13^C_ts,_ from −0.46‰ to 1.35‰ for Δδ^13^C_f‐g_ and from 0.49‰ to 2.3‰ for Δδ^13^C max. These samples were classified as pure honey also using EA‐IRMS official AOAC Method 998.12. The mean δ^13^C values of authentic honey are summarized in Table 4 and its chromatographic profile is shown in Figure 3. It is evident that the δ^13^C value of disaccharides is more negative than the δ^13^C value of glucose and the δ^13^C value of fructose by factor of 0.7‰, a little bit lower than the differences (1.2 and 1.3‰, respectively) reported by Cabañero et al.^22^

**TABLE 4:** Summary for δ^13^C values in fructose (δ^13^C_f_), glucose (δ^13^C_g_), disaccharides (δ^13^C_ds_), and trisaccharides (δ^13^C_ts_) in 19 authentic honey samples

Sugar content	Mean ± SD (‰)	Range (‰)^21^	Range (‰)^26^	Range (‰)^28^
δ^13^C_f_	−25.2 ± 0.8	−23.2 to −27.5	−23.14 to −27.29	−24 to −26.8
δ^13^C_g_	−25.2 ± 0.8	−22.7 to −27.2	−22.89 to −27.12	−24.2 to −27
δ^13^C_ds_	−26 ± 0.8	−22.5 to −28.2	−23.3 to −27.87	−24 to −28.8
δ^13^C_ts_	−25.5 ± 1.8	−22.6 to −27.5		−22.8 to −27.8

Six honey samples (S1, S3, S4, S5, S6, and S31) had Δδ^13^C_f‐g_ and Δδ^13^C max outside the natural honey range. Furthermore, these samples had δ^13^C values of individual sugars outside the acceptable ranges reported by Elflein and Raezke; so, they were classified as honey adulterated by C4 sugar. These results were also confirmed by the relative amount of oligosaccharides in honey (Figure 2) at percentage area higher than 0.7% (Table 3). It is also noticeable that δ^13^C_g_ values for those samples are close to δ^13^C_h_ values obtained by EA‐IRMS. This finding may reveal that honey samples were adulterated mainly with glucose sugar.

Seven samples (S2, S15, S19, S20, S28, S30, and S33) showed Δδ^13^C max higher than 2.1‰ and Δδ^13^C_f‐g_ values less than ±1‰. S15 was classified as authentic by AOAC official method 998.12 when we take into account the associated uncertainty with apparent C4 sugar content of 8.6%. By applying the LC‐IRMS method the sample showed a Δδ^13^C max value of 2.81‰, which falls outside the authentic honey range, indicating that this sample was manipulated. Samples, S15, S28 and S30 had high Δδ^13^C max values of 2.81‰, 7.38‰, and 2.95‰, respectively, between δ^13^C_ts_ and δ^13^C_p_, whereas sample S33 had high Δδ^13^C max value of 2.92‰ between δ^13^C_f_ and δ^13^C_p_. The highest Δδ^13^C max values of sample S19 and S20 reached 3.64‰ and 3.28‰, respectively, between δ^13^C_g_ and δ^13^C_ts_. These results show the importance of considering all the possible differences between the individual δ^13^C values of sugars including trisaccharides and δ^13^C values of proteins for the detection of honey adulteration. Based on the purity criteria of Elflein and Raezke, a honey sample is considered as non‐compliant if one of the ∆δ^13^C falls outside the limit. It should be noted here that both Δδ^13^C max and relative peak area of the oligosaccharide in sample S19 and S20 were non‐compliant with the purity criteria in honey. This was most probably due to an adulteration with a mixture of C3 and C4 sugars at levels >20%.^19^ Therefore, the samples (S15, S19, S20, S28, S30, and S33) were classified as adulterated honeys.

## 4 CONCLUSION

By analyzing δ^13^C values of bulk honey and its proteins using EA‐IRMS and δ^13^C values of individual sugars (fructose, glucose, disaccharides, and trisaccharides) using LC‐IRMS in 33 Lebanese honey samples, 20 samples were classified as authentic and 13 as adulterated honey. Out of 20 authentic samples, 18 samples were from beekeepers and 2 from the market. This indicates that beekeepers are the most reliable source to obtain authentic honey.

Among the 13 adulterated samples, 8 did not comply with all the limits defined by White et al. (δ^13^C_h_ < −23.5‰), by AOAC 998.12 (Δδ^13^C_p‐h_ < 1‰), and by Elflein and Raezke (Δδ^13^C max and Δδ^13^C_f‐g_ < ±2.1‰ and ±1‰, respectively). Four samples had δ^13^C_h_ < −23.5‰, but Δδ^13^C_p‐h_ > 1‰ and Δδ^13^C max > ±2.1‰. One adulterated sample complies for δ^13^C_h_ and Δδ^13^C_p‐h_ but its Δδ^13^C max exceeded the naturally occurring range of 2.1‰. The oligosaccharide peak was detected in most adulterated honey samples (n = 9) at a relative area higher than 0.7%. These results show the importance of considering all the possible differences between the individual δ^13^C values of sugars including trisaccharides and δ^13^C values of proteins for the detection of honey adulteration through illegal addition of sugar.
